# Pharmacokinetic profile of oral firocoxib in the koala (*Phascolarctos cinereus*)

**DOI:** 10.1371/journal.pone.0332448

**Published:** 2025-09-30

**Authors:** Amanda June Shapiro, Benjamin Kimble, Frances Hulst, Kimberly Vinette Herrin, Caroline Marschner, Chien-Jung Chen, Merran Govendir

**Affiliations:** 1 Sydney School of Veterinary Science, The University of Sydney, Sydney, Australia; 2 Taronga Conservation Society Australia, Mosman, Sydney, New South Wales, Australia; China Academy of Chinese Medical Sciences Institute of Chinese Materia Medica, CHINA

## Abstract

The pharmacokinetic profile of firocoxib administered orally at 5 mg/kg as a single dose to three mature koalas of each sex is described. After single dose administration, the harmonic means for maximal plasma concentration (C_max_), time to reach C_max_ (T_max_) and elimination half-life (t_1/2_) were 79.2 ng/mL, 3.69 h, and 5.01 h, respectively. Males exhibited a higher C_max_ than females (217.4 vs 48.4 ng/mL), suggesting greater oral absorption, while females had a slower elimination (t_1/2_: 5.87 h vs 4.38 h in males), however these sex differences were not significant. After a wash-out period, the same dose was administered every 12 h, on six occasions to the same koalas. With repeat dosing, females had higher trough concentrations at 24 and 48 h, and 12 h after the last dose, compared to the males. No observable adverse effects were evident in any of the koalas following repeat dosing. The target plasma concentration was approximately 100 ng/mL based on *in vitro* studies in other species to inhibit 80% of cyclooxygenase-2 activity (inhibitory concentration 80 [IC80]). During single firocoxib administration, this plasma concentration was attained only in the males and not in the females. Two unidentified metabolites were also observed in the chromatograms. Acknowledging the lipophilic nature of firocoxib and that it is therefore likely to persist in animals’ tissues longer than in plasma, firocoxib appears a useful nonsteroidal anti-inflammatory drug for the koala. The oral dose of 5 mg/kg twice a day for a maximum of six doses appears safe for mature male koalas. The data presented here suggests that mature females may require a higher dose (perhaps double the dose i.e., i.e., 10 mg/kg). Until further studies confirm the sex differences in pharmacokinetic profile of firocoxib and safety of higher doses in female koalas, a once daily administration of a higher dose is suggested.

## Introduction

The koala is an iconic Australian marsupial, and classified by the Australian Government as endangered in the states of New South Wales and Queensland, and in the Australian Capital Territory [1]. Free range koalas are exposed to threats of injury and death that include severe weather conditions (bush fires and heat waves), vehicle strikes, feral animal and dog attacks, and infectious diseases such as chlamydiosis [2]. As a result, injured koalas require rescue, treatment and rehabilitation, the success of which depends on efficacious analgesic and anti-inflammatory medicines.

Over the past decade there have been pharmacokinetic (PK) profiles published for some analgesics for the koala. These include opioid or opioid-like analgesics, e.g., tramadol as a subcutaneous (s.c.) bolus injection [3]; and fentanyl as an intravenous bolus injection, or as a constant rate infusion, or applied as a 25 µg/h transdermal patch [4]. The pharmacokinetic profile of the non-steroidal anti-inflammatory drug (NSAID) meloxicam for the koala is available [5]. Meloxicam has poor oral absorption in the koala and when administered intravenously, has a short elimination plasma half-life of only 1.19 h (range 0.71 to 1.62 h) [5] compared with 24 h in dogs [6] and approximately 13 h in humans [7]. Therefore, in contrast to many other species, meloxicam would require frequent dosing which is problematic from the management side and stressful for a non-domesticated species. When carprofen was incubated with koala hepatic metabolism enzymes, the intrinsic clearance was rapid, suggesting that a practical efficacious daily or twice daily dosing regimen in the live animal could be problematic [8]. The most recently investigated analgesic for koalas is paracetamol (also known as acetaminophen), which shows promise as a component of a multi-modal analgesic plan [9]. The search for an efficacious NSAID for the koala that can be dosed twice daily or less frequently, is continuing.

NSAIDs produce analgesic and anti-inflammatory effects by inhibiting the enzyme cyclooxygenase (COX), that inhibits the synthesis of inflammatory and pain inducing prostaglandins [10]. Cyclooxygenase exists in two isoforms, COX-1 and COX-2, and selective COX-2 inhibitors are more gastroprotective than non-selective COX inhibitors and preferential COX-2 inhibitors [11]. Firocoxib, a selective COX-2 inhibitor, is a veterinary NSAID, only available as tablets. It is registered for control of pain and inflammation associated with osteoarthritis in dogs and horses, and postoperative pain and inflammation associated with orthopaedic and/or soft tissue surgery in dogs. The dosage in dogs and horses is 5 mg/kg and 0.1 mg/kg, respectively, every 24 h. Currently there are no published studies on the pharmacokinetic profile of firocoxib in koalas. Therefore, the overall objective of this study was to describe the pharmacokinetic profile of firocoxib after a single oral dose in mature, clinically normal koalas, followed by repeated dosing every 12 h after six doses to ascertain whether there was any drug accumulation and to monitor for any adverse drug events.

## Materials and methods

### Animals

Six mature koalas (3 males, 3 females) with median (range) body weights and ages of 9.2 kg, (7.5 to 9.7 kg) and 4.8 years (4.3 to 9.1 years), respectively, were recruited from the Taronga Zoo colony (Mosman, NSW, Australia). These koalas were clinically normal based on regular physical examinations, haematological and biochemical analyte values. During the study, koalas were housed singly and supplied with various *Eucalyptus* spp. foliage and water *ad-libitum*. This study was approved by the Taronga Conservation Society of Australia, Animal Ethics Committee protocol 3a/04/23. No animals were sacrificed; the animals were returned to the colony immediately after firocoxib administration and blood sampling. In order to minimise stress, intravenous catheterisation for sequential blood sampling was performed under general anaesthesia.

### Blood collection and drug administration

#### Placement of intravenous catheter for blood collection.

The day prior to drug administration and blood collection, the koalas were anaesthetised with alfaxalone (Alfaxan, Jurox, Pty Ltd, Rutherford, NSW, Australia) at 3 mg/kg administered intramuscularly (i.m.) and maintained under anaesthesia on isoflurane in 100% oxygen via a face mask for placement of a 20 gauge, 1 ¼ inch intravenous catheter into the cephalic vein. A short T-connector extension set (Codan, Santa Ana, CA, USA) and cap was attached to the catheter and flushed with saline. The extension tube, cap and catheter were secured with tape and bandage, for serial blood collection. Blood (5 mL) was collected at the time of anaesthesia in a lithium heparin tube to establish a baseline (t = 0 h).

### Dosing of animals, blood collection timepoints and sample handling

#### Single oral dose administration.

Firocoxib was administered as a single oral dose of 5 mg/kg as crushed tablets made into a paste with Vetafarm Koala Crittacare (Wagga Wagga, NSW) and water. Blood (up to 2 mL) was collected into lithium heparin tubes at t = 0.25, 0.5, 1, 2, 4, 8, 12, 24, 36 and 48 h after firocoxib administration.

#### Repeated oral dosing.

At least 2 months after the initial single oral dose administration study, firocoxib was administered twice daily at a dose of 5 mg/kg orally (as described in the single dose administration) for three consecutive days with the last dose administered 60 h after first administration, with a total of six doses administered. Blood (2 mL) was collected once daily into lithium heparin tubes at 24 and 48 h after first firocoxib dosage (prior to the morning dose). The cap, extension tube and catheter were flushed with saline before and after each collection. The first 0.5 mL of blood was discarded to avoid dilution of samples with saline.

Samples were centrifuged at 1500 *x g* for 10 minutes, within 1 h of collection; the plasma was immediately stored at –80 °C and protected from light until drug quantification. Blood was collected in EDTA tubes and in serum tubes at t = 0 and t = 72 h from two koalas to detect changes in haematology, biochemical analytes and electrolytes. Clinical observations for the presence of adverse effects in the animals treated included measurement of vital signs, appetite, body weight, faecal consistency and overall behaviour and mobility.

### Drug analysis method and sample processing

#### Chemicals.

The following chemicals were acquired for this study: firocoxib ≥ 98% purity (Cayman Chemical, CAS # 189954-96-9, MI, USA), high pressure liquid chromatography (HPLC) grade acetonitrile, ethyl acetate, ammonium acetate, triethylamine and acetic acid were purchased from Thermo Fisher Scientific (Macquarie Park, NSW, Australia).

#### Drug analysis.

Quantification of firocoxib and visualisation of metabolites in plasma was achieved by HPLC with fluorescence detection modified from a published method [12]. The HPLC system consisted of a Shimadzu LC-20AT solvent delivery unit, DGU-20A degasser, a SIL-20A HT auto injector, an SPD-20A UV detector and RF-10A XL fluorescence detector (Shimadzu, Rydalmere, NSW, Australia) and column oven (CTO-20A). Shimadzu LC solutions software, LabSolutions version 5.98 (Kyoto, Japan) was used for chromatographic control, data collection and data processing. Chromatographic separation was performed with a Supelco, Discovery C18, (25 cm x 4.6 mm, 5 µm) column (Merck KGaA, Darmstadt, Germany) with a 1-mm Opti-guard C18 pre-column (Choice Analytical, Thornleigh, NSW, Australia) at ambient temperature. The isocratic mobile phase consisted of 20 mM ammonium acetate buffer and acetonitrile (55:45, v/v), 0.25% acetic acid, 0.1% triethylamine adjusted to pH 4.5 with acetic acid. The total run time was 12 min, at a flow rate of 1.2 mL/min. Fluorescence detection excitation and emission wavelengths were 280 nm and 375 nm, respectively.

For sample preparation 200 μL of each of the following: plasma samples collected from koalas administered firocoxib, standards prepared for the standard curves and the QC samples; were extracted twice by mixing with 800 μL of ethyl acetate. The extracted organic portion (1.6 mL) was dried under vacuum in a Speed Vac Concentrator (Thermo Scientific, MA, USA) at 35 °C for 1 h, reconstituted with 100 μL of acetonitrile, and finally 10 μL of the reconstituted sample was injected into the HPLC system.

External standard curve concentrations using weighting factor (1/*x*^*2*^), ranging from 15.63 to 500 ng/m (all with *r*^2^ > 0.99), and quality control samples were prepared in blank pooled koala plasma (n ≥ 3). The lower limit of quantification (LLOQ) for firocoxib was determined by an external standard comprising three known concentrations of firocoxib (250 mg/mL, 62.5 ng/mL, 15.63 ng/mL). Based on the lowest concentration where the precision and accuracy were <15% and within 20% of nominal concentration, the LLOQ for firocoxib was calculated as 15.6 ng/mL. This LLOQ was well above the theoretical LLOQ calculated from intercept (SD) and slope (average) of regression lines and met the criteria for setting the LLOQ [13]. The concentration of each QC sample was repeated three times with the accuracy and precision calculated on each of the triplicates. The mean accuracy ± standard deviation (SD) were calculated using the formula [13]:


$$Accuracy(\%)=\frac{EstimatedQCconcentration}{ActualQCconcentration}x\text{1}\text{0}\text{0}$$

Precision (CV %) was obtained by dividing the standard deviation by the mean estimated concentration, multiplied by 100 [13]. The mean accuracy as percent relative error (%RE) and precision as percent coefficient of variation (%CV) with standard deviation of the quality control samples was 3.4% ± 6.9% and 13.8% ± 12.7%, respectively.

The recovery rate of firocoxib from koala plasma was assessed by comparing the detector responses of firocoxib spiked koala plasma, in terms of area under the curve, to pure standard dilutions of firocoxib in acetonitrile within the calibration range. The mean recovery of firocoxib calculated was 90% ± 1.4%.

#### Pharmacokinetic analysis.

Firocoxib plasma concentrations from the initial oral administration underwent non-compartmental analysis. The peak concentration (C_max_) and the time reached (T_max_) were obtained directly from the measured concentrations. The elimination half-life (t_1/2_) was determined by ln2/k_e_ where k_e_ is the elimination rate constant which is the inverse slope of the elimination or terminal part of the semi-log curve. The absorption constant k_a_ was determined by the method of residuals [14]. The area under the concentration-time curve (AUC_0–24_) was calculated to the measurable concentration at 24 h (time ‘t’ in equations) using the log-linear trapezoidal method [15]. The AUC and AUMC from the last observed concentration to infinity were determined by


$$AUC_{t-\infty }=\frac{C_{\text{1}ast}}{k_{e}}$$

$$AUMC_{t-\infty }=\left(C_{\text{1}ast}\times \frac{t_{last}}{k_{e}}\right)+\left(\frac{C_{last}}{k_{e}^{\text{2}}}\right)$$

The mean residence time (MRT) was determined by the following equation:


$$MRT=\frac{{AUMC}_{\text{0}-\infty }}{{AUC}_{\text{0}-\infty }}$$

PK Solver [16] was used to undertake the noncompartmental analysis and k_a_ calculation.

As this was an oral study only, with no IV dosing, the absolute oral bioavailability (F) could not be determined. Therefore, the steady state volume of distribution/ F [V _ss_/F] was calculated

as $\frac{dosexAUMC}{{AUC}^{\text{2}}}$

The volume of distribution area/ F [V_z_/F] was calculated as


$$\frac{dose}{{AUC}_{\text{0}-\infty }xk_{e}}$$

and the clearance/ F [CL/F] = $\frac{dose}{{AUC}_{\text{0}-\infty }}$ [17].

With the repeated oral dosing, the accumulation factor equation [18] was used to ascertain if there was any firocoxib accumulation between trough concentrations at 48 h versus 72 h:


$$Accumulationfactor=\frac{\left(\text{1}-{\text{e}}^{-nkT}\right)}{\left(\text{1}-{\text{e}}^{-kT}\right)}$$

Where n = the number of doses

k = k_e_ = elimination constant

τ = number of hours between dosing [18]

#### Firocoxib binding to plasma proteins.

The percentage of firocoxib bound to koala plasma proteins was determined using the ultrafiltration method [19] using a modified protocol [5]. As firocoxib plasma protein binding (PPB) has been reported in horses [20], archived horse plasma (obtained under The University of Sydney approved animal ethics protocol: 2015/736) was tested simultaneously alongside koala plasma to compare the percentage of firocoxib bound to equine plasma proteins. The total plasma protein concentration in the koala samples was determined using Vetscan® VS2 chemistry analyser (Comprehensive Diagnostic Profile), (Zoetis Australia Pty Ltd., Rhodes, NSW). Firocoxib dissolved in dimethyl sulfoxide (to reduce non-specific binding) at a concentration of 500 ng/mL, was added to 1 mL of thawed pooled blank koala or horse plasma, the pH was adjusted to 7.4, and then incubated in a water bath at 37 °C for 30 min. Then 100 μL of plasma was removed for determination of the total firocoxib concentration (Drug _total_) and the remaining plasma was transferred to the reservoir of the Centrifree® ultrafiltration device (Merk Millipore, Macquarie Park, Australia) with a membrane molecular weight cut‐off of 30 kDa. The ultrafiltration device was centrifuged at 1500 x *g* for 30 min at 37 °C [19]. After centrifugation, the filtrate was used to determine the free firocoxib concentration (Drug _free_). Both fractions were analysed by HPLC as described above. All samples were analysed as duplicates. The percentage of substrate binding to plasma proteins was calculated using the equation:


$$\%\text{PPB}=\left\{\frac{{Plasma}_{total}-{Plasma}_{free}}{{Plasma}_{total}}\right\}x\text{1}\text{0}\text{0}$$

The same concentrations of firocoxib were added to phosphate buffered saline and underwent ultrafiltration to determine non-specific binding to the filtration membrane. Thereafter the corrected percentage of substrate binding to plasma proteins was calculated as:


$${\%PPB}_{\left(NSBcorrected\right)}=\left\{\frac{[\frac{{Buffer}_{free}}{{Buffer}_{total}}-\left[\frac{{Plasma}_{free}}{{Plasma}_{total}}\right]}{\left[\frac{{Buffer}_{free}}{{Buffer}_{total}}\right]}\right\}x\text{1}\text{0}\text{0}$$

[21].

### Statistical analyses

The single dose AUC_0–24_ for firocoxib; for metabolite M1; and the repeated dosing firocoxib concentrations at 24, 48 and 72 h between the females versus the males, were compared using a t test with a Welch’s correction that did not assume equal standard deviations.

For the repeat dosing, a repeated measures two-way ANOVA was used to investigate whether there was a sex or time effect on firocoxib concentrations at 24, 48 and 72 h. For all statistical analyses, the level of significance was p < 0.05. The data was analysed using Prism 10.4.1 (GraphPad Software, San Diego, CA, USA). Due to the great variation in pharmacokinetic indices, not only are the arithmetic means, standard deviations and median and ranges provided but also the harmonic mean as this gives a more conservative measure of central tendency [22].

## Results

The weights, sexes and ages of all koalas are provided in S1 Table. Table 1 provides firocoxib plasma concentrations at all time points in all six koalas.

**Table 1:** Firocoxib plasma concentrations (ng/mL) at each blood collection time-point for each koala, when 5 mg/kg of firocoxib was administered as a single oral dose.

Time (h)	K1 (Male)	K2 (Male)	K3 (Male)	K4 (Female)	K5 (Female)	K6 (Female)
0	0	0	0	0	0	0
0.25	7.94	4.37	16.6	0.78	3.74	3.58
0.5	4.51	10.0	52.5	1.23	5.88	10.6
1	8.34	21.7	41.9	8.27	28.6	7.89
2	22.7	39.2	105.2	24.3	**61.9**	12.3
4	68.3	**210.1**	**263.4**	**89.3**	51.8	**28.9**
8	**190.8**	117.6	127.8	74.7	45.8	17.5
12	100.9	39.7	49.6	40.2	23.3	13.2
24	57.2	4.13	3.86	3.70	12.8	3.78
36	ND	ND	ND	ND	3.83	7.02
48	ND	ND	ND	ND	ND	3.83

Cmax value for each koala is bolded. Grey cells denote firocoxib concentrations greater than 100 ng/ml.

ND = not detectable.

### Pharmacokinetic analysis

The individual pharmacokinetic (PK) indices for each of the six koalas for the single oral dose of 5 mg/kg is provided in Table 2.

**Table 2:** Individual pharmacokinetic indices of firocoxib, when 5 mg/kg of firocoxib was administered as a single oral dose to the six koalas.

Pharmacokinetic indices	K1	K2	K3	K4	K5	K6
k_a_ (1/h)	0.11	0.21	0.17	0.43	0.25	0.25
k_e_ (1/h) ke slope was calculated using concentrations at these points (h)	0.05 12, 24	0.21 4, 8, 12	0.22 4, 8, 12	0.19 8, 12, 24	0.07 8, 12, 24	0.10 8,12,24
t_1/2_ (h)	14.7	3.31	3.19	3.67	9.91	7.24
T_max_ (h)	8.00	4.00	4.00	4.00	2.00	4.00
C_max_ (ng/mL)	190.8	210.1	263.37	89.3	61.86	28.90
AUC_0-t_ (ng/ml*h)	2163.0	1522.8	1934.5	953.6	820.07	443.8
AUC_0-inf_ (ng/ml*h)	2995.1	1543.5	1952.3	973.1	868.21	537
AUC _0-t_/AUC_0-inf_	0.72	0.99	0.99	0.98	0.94	0.83
AUMC _0-inf_ ng/mL*h^2^	56998	11422	13436.9	8500.1	11402.2	14183.2
MRT_0-inf_ (h)	19.0	7.13	6.88	8.74	13.13	26.41
Vz/F (L)	276.6	152.1	113.1	202.5	615.7	2128.8
Cl/F (L/h)	19.0	30.5	24.6	38.5	49.2	87.5

Table abbreviations: k_e _= elimination constant = slope of the elimination phase; t_1/2 _= elimination half-life; T_max_ = time to reach maximal plasma concentration; C_max_ = maximal plasma concentration; AUC_0-t _= log linear area under the firocoxib plasma concentration time curve form 0 to last measured concentration; AUC_0-inf _= log linear area under the firocoxib plasma concentration time curve from 0 h to infinity h; AUMC_0-t/0-inf_ = area under the moment curve from 0 – inf; MRT = mean residence time; Vss/F = volume of distribution at steady state relative to the amount of drug absorbed, Vz/F = volume of distribution during elimination relative to the amount of drug absorbed; Cl/F = drug clearance relative to the amount of drug absorbed

**Fig 1:**
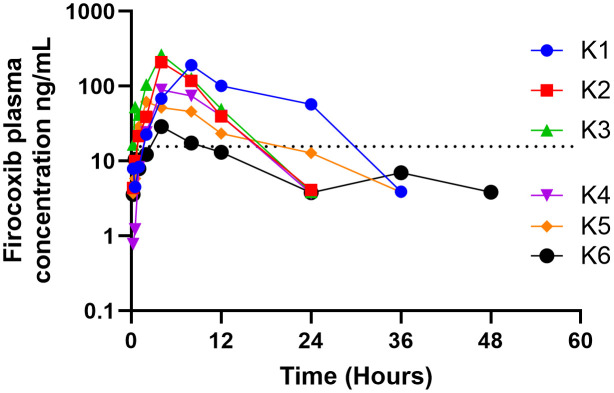
Firocoxib semi-log plasma concentration versus time curves after oral administration (*n* = 6) of firocoxib (5 mg/kg) in clinically normal koalas. The dotted horizontal line represents the LLOQ (15.6 ng/mL).

A summary of this data presented as the mean ± SD, median and range for all six koalas is provided in Table 3. The semi-log firocoxib concentration versus time curves for the single oral administration to each of the six koalas are presented in Fig 1.

**Table 3:** Pharmacokinetic indices of firocoxib when administered orally, once at 5 mg/kg (n = 6). Data provided as harmonic and arithmetic means + /- SD, median, range and coefficient of variation (CV%).

Indices	Harmonic mean ng/mL	Arithmetic Mean ng/mL	SD ng/mL	Median (range) ng/mL	CV %
**k**_**a**_ **(1/h)**	0.20	0.24	0.10	0.23 (0.11-0.43)	41.9
**k**_**e**_ **(1/h)**	0.10	0.14	0.07	0.15 (0.05 - 0.22)	48.9
**t**_**1/2**_ **(h)**	5.01	7.00	4.33	5.46 (3.17 - 14.7)	43.3
**Tmax (h)**	3.69	4.33	1.80	4.00 (2.00 - 8.00)	41.4
**Cmax (ng/mL)**	79.2	140.7	85.4	140.1 (28.9 - 263.4)	60.7
**AUC**_**0-t**_ (ng/ml*h)	974.4	1306.5	66.5	1238.22 (443.8–2163)	47.2
**AUC**_**0-inf**_ (ng/mL*h)	1083.9	1478.2	820.9	1258.3 (537.0–2995.1)	55.5
**AUC 0-t/0-inf**	0.90	0.91	0.10	0.96 (0.72 - 0.99)	11.1
AUMC ng/mL*h ^**2**^	13176.1	19323.8	16944.9	12429.4 (8500.1–56998.2)	87.7
**MRT**_**0-inf**_ **(h)**	10.7	12.0	4.51	12.22 (7.09–18.91)	37.6
**Vss/F (L)**	332.3	673.5	727.5	811.2 (890.2–931.0)	107.7
**Vz/F (L)**	230.3	581.5	711.3	239.6 (113.1–2128.8)	122.3
**Cl/F (L/h)**	32.6	41.6	22.7	34.5 (19.0-87.5)	54.7

Table abbreviations: k_a_ = absorption constant, k_e _= elimination constant = slope of the elimination phase; t_1/2 _= elimination half-life; T_max_ time to reach maximal plasma concentration; C_max_ = maximal plasma concentration; AUC_0–24 _= log linear area under the firocoxib plasma concentration time curve form 0–24 h; AUC_0-inf _= log linear area under the firocoxib plasma concentration time curve from 0 h to infinity h; AUC_0-t/0-inf_ = area under the moment curve from 0-t/0-inf h after firocoxib dosing; MRT = mean residence time. Vss/F = volume of distribution at steady state relative to the amount of drug absorbed, Vz/F = volume of distribution during elimination relative to the amount of drug absorbed; Cl/F = drug clearance relative to the amount of drug absorbed

Table 4 provides the mean male and female plasma firocoxib PK indices for the single dose study.

**Table 4:** Pharmacokinetic indices of firocoxib, when 5 mg/kg of firocoxib was administered as a single oral dose to the three male versus the three female koalas.

	Male (n = 3)				Female (n = 3)			
Pharmacokinetic indices	Harmonic mean ng/mL	Arithmetic mean ng/mL	SD ng/mL	CV %	Harmonic mean ng/mL	Arithmetic mean ng/mL	SD ng/mL	CV %
k_a_ (1/h)	0.16	0.19	0.06	31.0	0.25	0.28	0.11	38.4
k_e_ (1/h)	0.10	0.16	0.08	48.5	0.10	0.12	0.05	42.5
t_1/2_ (h)	4.38	7.06	5.4	76.5	5.87	6.94	2.56	36.8
T_max_ (h)	4.80	5.33	1.89	35.4	3.0	3.33	0.94	28.3
Cmax (ng/mL)	217.4	221.4	30.68	13.9	48.4	60.3	24.7	41.1
AUC_0-t_ (ng/ml*h)	1833.8	1873.4	264.9	14.1	663.5	739.1	215.9	29.2
AUC _0-inf_ (ng/ml*h)	2008.3	2163.6	611.2	28.2	742.3	792.8	185.9	23.4
AUC _0-t_/AUC_0-inf_	0.88	0.90	0.13	14	0.91	0.92	0.07	7.2
AUMC _0-inf_ ng/mL*h^2^	16711.5	27285.7	21026.0	77.1	10875.3	11361.8	2320.3	20.4
MRT_0-inf_ (h)	9.01	11.1	5.61	50.5	13.1	16.09	7.51	46.7
Vz/F (L)	157.6	180.6	69.7	38.6	436.7	982.4	828.0	84.3
Cl/F (L/h)	23.8	24.7	4.7	18.9	52.0	58.4	21.0	36

The individual trough firocoxib concentrations for each of the six koalas for the repeated oral dose of 5 mg/kg twice daily (bid) is provided in S2 Table. A summary of this data, presented as the mean ± SD, median, range, and coefficient of variation, including the accumulation factor at 72 h compared to 48 h, for all six koalas is provided in Table 5. While S3 Table provides male versus female plasma firocoxib trough concentrations for the repeated dosing study.

**Table 5:** Harmonic mean, arithmetic mean ± SD (ng/mL), median and range (ng/mL) and CV (%), of firocoxib plasma trough concentrations at blood collection time points just prior to the next dose, and at 72 h which was 12 h after the last dose. The accumulation factor using trough concentrations at 48 h versus 72 h for each koala are also provided.

		ng/mL					
Time (h)	Harmonic mean	Arithmetic mean	SD	Median	Min	Max	CV %
**24**	43.3	54.7	26.1	58.3	25.3	88.9	47.6
**48**	33.2	47.4	20.6	52.9	11.9	69.6	43.6
**72**	68.8	85.5	40.0	82.1	35.9	136.7	46.7
		K1	K2	K3	K4	K5	K6
**Accumulation factor**		1.75	1.09	1.09	1.43	1.75	1.43

**Fig 2:**
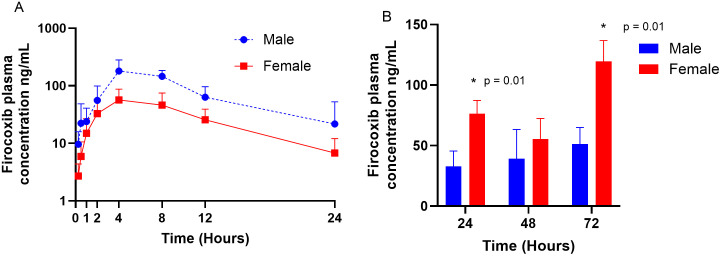
Firocoxib plasma concentrations over time for single dose (A) and repeat dosing (B). A: Firocoxib plasma concentrations after single dose presented as mean ± SD, male versus female and B: mean ± SD trough concentrations after repeat dosing at 24, 48 and 72 h. *Denotes significantly different means between males and females at that timepoint.

For the single administration, the male mean ± SD AUC_0–24_ appeared greater than that of the females, however there was no statistical difference (p = 0.08) (Fig 2A.). Additionally, there were no significant differences between the mean ± SD firocoxib trough concentrations between 24, 48 or 72 h within the female, or within the male koalas. However as illustrated in Fig 2B, the mean ± SD trough concentrations at 24 h (just prior to the third dose), 48 h (just prior to the fifth dose) and at 72 h (12 h after the last dose) were always greater for the females than the males. A 2-way ANOVA demonstrated a significant difference in the firocoxib mean ± SD between the male verses the female koalas at 24 and 72 h, both p = 0.01, but not at 48 h. The accumulation factor for all six koalas between 24 and 48 h is provided in Table 2 and four koalas (1 male and all three females) had an accumulation factor > 1.3 which signifies moderate drug accumulation [22].

The corrected percentage of firocoxib (at a concentration of 500 ng/mL) bound to koala and horse plasma proteins was 84.7 and 85.3%, respectively, when taking into account the non-specific binding of firocoxib (at a concentration of 250 ng/mL) of 4.06%.

**Fig 3:**
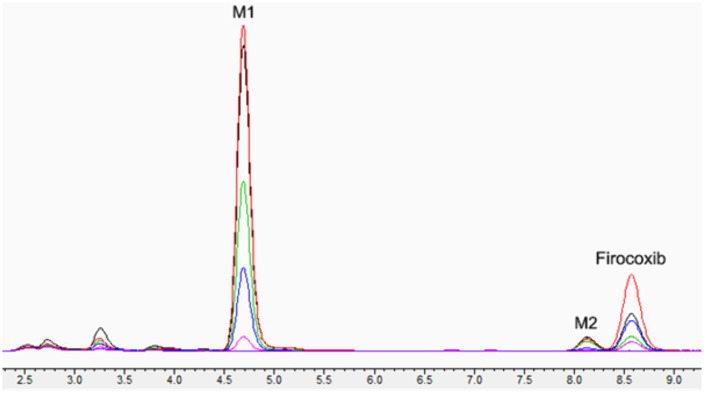
Superimposed chromatograms of K3’s plasma samples. X axis is time in min, Y axis is millivolts (mV). Metabolite M1 is observed at approximately 4.7 min showing amplitude changing with time (purple trace = 0 h pink trace = 1 h, blue trace = 2 h, red = 4 h, black = 8 h, green = 12 h); Firocoxib peak is at 8.6 min, and metabolite M2 is observed at approximately 8.1 min.

Two metabolites were evident on the chromatogram demonstrated in Fig 3. The retention time of firocoxib was approximately 8.6 min with two metabolites observed. The metabolite with the shortest retention time at approx. 4.7 min is designated as M1, the metabolite with a retention time of approximately 8.1 min, is designated as M2.

The change in the area under the chromatogram peaks for M1 and M2 for each koala for the single dose study is provided in S4 and S5 Tables, respectively.

**Fig 4:**
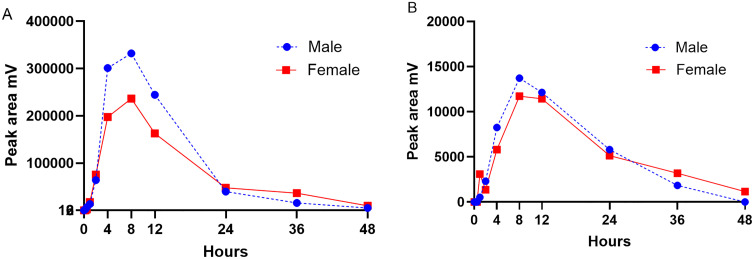
Mean ± SD change in metabolites M1 (A) and M2 (B) chromatogram peak amplitude versus time, males compared to females after a single dose of firocoxib.

The change in the metabolites’ amplitude over time in the single dosing study between males versus females is illustrated in Fig 4. There was no significant difference in the AUC_0-48h_ of M1 or M2 between the males and females.

**Fig 5:**
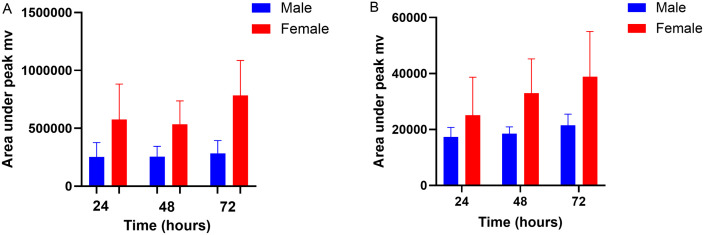
Mean ± SD change in metabolites M1 (A) and M2 (B) chromatogram peak amplitude over time, males compared to females after a repeat dosing of firocoxib.

The change in the area under the chromatogram peaks for M1 and M2 for each koala for the repeat dose trough levels are provided in S6 and S7 Tables, respectively. The change in the metabolites’ (M1 and M2) chromotogram peak amplitude over time in the repeat dosing study between males versus females is illustrated in Fig 5. There were no significant differences in the amplitude of M1 and M2 at each timepoint between the sexes.

Full blood counts and plasma biochemistry was performed prior to dosing and at 72 h after the repeat dosing for two koalas only. There were no significant changes in any indices between 0 and 72 h. This data is provided in S8 Table.

## Discussion

As a 5 mg/kg single oral dose, firocoxib could be detected in the plasma of all koalas after 15 min and reached a harmonic mean C_max_ = 79.2 ng/mL at 3.69 h. When the recommended oral dose of 5 mg/kg is administered to dogs, the mean C_max_ is 520 ng/mL [23], whereas 0.1 mg/kg administered orally to horses, has a C_max_ of 58 ng/mL [24]. Of the three species, koalas had the longest T_max_, followed by horses (mean 3.2 h) [24], with dogs having the fastest T_max_ (1.25 h) [25]. In this study firocoxib concentrations were not detectable at 36 h in four of the koalas (three males, one female) although concentrations in the other two females were detectable, but below the LLOQ. The absorption phase (k_a_) was faster than the elimination phase (k_e_) in all koalas. The harmonic mean plasma elimination half-life in the koala (5.01 h) is comparable to dogs (mean 5.90 h) [25] and considerably shorter than in horses (32.8 h) [24]. A summary of firocoxib C_max_, T_max_ and half-life when administered intravenously or orally in other species is provided in S9 Table. It is recognised that although NSAIDs can have a short plasma half-life, concentrations in tissues and inflammatory exudates can persist much longer than plasma concentrations [10]. The MRT which indicates the average time that the drug molecule remains in the body [22], has a harmonic mean of 10.7 h in the koala.

Table 1 demonstrates substantial variation (standard deviation and coefficient of variation) among many PK indices. Differences in oral absorption among individuals may account for some variation. Eucalypt leaf ingesta is a constant feature within the lumen of the koala gastrointestinal tract (git) and dependent on the amount and state of ingesta in the individual’s git, may entrap varying proportions of the firocoxib. The sex of the animal may also have contributed to indices variation as the AUC_0–24_ was greater in the males than females after a single dose (Fig 2A), although this difference was not statistically significant. Substantial variability in some firocoxib pharmacokinetic indices have also been reported in beagles of both sexes with no mention of sex-specific differences only large inter-animal variation [26].

Absolute oral bioavailability in horses dosed at 0.1 mg/kg firocoxib was 79% [20] and in dogs administered firocoxib at 5 mg/kg oral bioavailability ranges from 16.6 to 78.7% [26]. Firocoxib was not administered by any parenteral route to the koalas, therefore absolute and relative bioavailability, Vd_ss_, Vd_area_ or Cl, could not be calculated. Firocoxib has a Vd of 1.7 L/kg in horses and tissue residue studies demonstrated wide tissue distribution, while the Cl was reported as 37 mL/h/kg [27].

In this study, firocoxib was administered at 5 mg/kg every twelve hours for six doses to koalas. An accumulation factor of approximately 1.1 was calculated for animals K2 and K3 (both males), but exceeded 1.3 for K1 (male) and K4, K5 and K6 (females) indicating some drug accumulation [22]. Additionally, there was a significant difference in the firocoxib trough concentrations between the males and females at 24 and 72 h (both p = 0.01) see Fig 2B. In those animals with an accumulation factor > 1.3, accumulation is likely avoided if firocoxib is administered once, rather than twice, daily.

Two metabolites, M1 and M2, were observed forming and then depleting in the chromatograms. In all koalas, M1 was detected from 1 to 48 h after firocoxib administration, while M2 was detected between 8 and 24 h. When horses were treated with firocoxib, the main metabolites identified were decyclopropylmethylfirocoxib (DFX) and firocoxib’s glucuronide conjugates [20]. When these metabolites were incubated with rat and horse hepatic microsomes and horse liver S9 fractions under oxidative conditions with phase 1 metabolism cofactors, the metabolites had low or no pharmacologic activity [20]. The metabolites in the koala plasma were not identified, future studies will endeavour to structurally characterise the observed metabolites using LC-MS/MS. However, the majority of NSAID metabolites tested have been found to be ineffective COX inhibitors [28]. The one metabolite identified in the rhinoceros firocoxib study (performed in this same laboratory with the same assay conditions) [12] differs in retention time to those observed in the koala plasma. After the single firocoxib dose the M1 peak AUC was greater (but not statistically significant) for the males than females (Fig 4A) but the M2 peak AUC did not appear to differ between the sexes (Fig 4B). Only C_max_, and not T_max_, was greater when firocoxib was administered to the males, suggesting possible increased firocoxib oral absorption which results in greater M1 generation. On the repeat dosing study there was a trend for the females to have higher trough concentrations of both M1 and M2, suggesting slower elimination of both compared to the males, but there were no significant differences between time point concentrations nor between sexes (Fig 5). In Fig 4A the elimination gradient appears steeper between 8–24 h in the males for M1 suggesting faster elimination of this metabolite. In S5 Table it appears that the CL/F is faster in the females than males, but this is deceptive as it is likely that the males have a higher oral bioavailability (i.e. F_oral_) that reduces Cl/F values.

All NSAIDs are usually highly bound (>90%) to plasma proteins and this is generally consistent between species [29]. This study attempted to confirm the proportion of firocoxib bound to koala plasma proteins. The ultracentrifuge method was used for repeated attempts on thawed, frozen koala plasma and horse plasma as a comparison [19]. Firocoxib (at a concentration of 500 ng/mL) was bound to koala and horse plasma proteins at 84.7 and 85.3%, respectively. Firocoxib has been previously reported as 97% bound in horse plasma [20], this is only one of two published methods on firocoxib plasma protein binding which detected radiolabelled firocoxib binding to equine plasma proteins. The second published method in African and Asian elephants using rapid equilibrium dialysis determined firocoxib binding to elephant plasma proteins to be 45% [30,31]. Firocoxib is a weak organic acid with a -log_10_ dissociation constant (pka) = 19.69 [32], resulting in low ionizability at physiological pH [33]. Firocoxib is considered insoluble in water but highly soluble in dimethyl sulfoxide [33]. The very high pka value may have affected the *in vitro* plasma protein binding assay results in this study as well as the difference in method compared to the only published protocol utilising radioactivity. It is preferable to use fresh plasma rather than thawed frozen plasma for investigating the medicine binding to plasma protein as the plasma protein structure can be altered by freezing [34] and this may have also affected the results reported in this study.

This study was conducted in clinically normal koalas and therefore no conclusions can be reached on the efficacy to control pain or inflammation in koalas. Firocoxib is the most highly COX-2 selective of all veterinary NSAIDs: 30-fold more potent than carprofen in COX-2 selectivity, with a COX-1:COX-2 inhibition ratio range of 384–427 in dogs [35] and a ratio of 643 in horses [33], both determined by COX inhibition assays in whole blood [36].

Firocoxib has been shown to have good efficacy in horses for controlling pain and inflammation associated with osteoarthritis [33]. Likewise, firocoxib has antipyretic activity in cats [37] and has been shown to have efficacy in improving the peak vertical force and weight-bearing capacity of dogs in an experimental model of urate crystal-induced synovitis [38]. As koalas and horses are both hindgut fermenters, there may be key similarities in firocoxib’s visceral analgesic effects in both species when presented with gastrointestinal colic, torsion or intussusception. In a study comparing the effects of firocoxib and flunixin meglumine on equine ischaemic intestinal injury, firocoxib provided superior mucosal barrier function recovery, while both NSAIDs provided efficacious visceral analgesia [39]. As a selective COX-2 inhibitor, firocoxib has been suggested as an equine neonatal visceral analgesic [40]. When used as a postoperative analgesic in ovariohysterectomised cats, a dose of 3 mg/kg/day, administered 1 h prior to induction of anaesthesia and at 24 and 48 h post-surgery, provided some analgesia however two out of eight cats developed a reversible azotaemia 72 h after surgery [41]. This finding in cats is congruent with a recent study which suggests that the use of NSAIDs in the postoperative period in cats may result in acute kidney injury [42].

In the horse, a plasma concentration exceeding 103 ng/mL is required to inhibit 80% of *in vitro* COX-2 reactions (i.e. corrected from the inhibitory concentration IC80 of 67 ng/mL in horse whole blood) [20,33,35,43]. This plasma concentration (although not a definitive therapeutic threshold for koalas) was achieved for 4 to –6 h in all three male koalas but was never reached in any female. Therefore, a suggestion is to double the dose in the females which should double the C_max_, however it is advisable that the frequency of administration should be reduced to once a day to prevent drug accumulation and the potential for adverse effects. For each animal, there was no change from normal appetite, behaviour and mobility during or after the study and all animals maintained stable body weight.

Like all veterinary patients, it is believed that pain management in koalas is crucial for recovery from injury and/or surgery, which also critically hinges on rapid return of appetite [44]. In koalas, with low body energy reserves, the rapid return of appetite is even more critical as they can quickly succumb following surgery or injury if they remain inappetent due to pain. As the elimination profile of firocoxib in the koala does not mirror that of other NSAIDs such as meloxicam [5] and carprofen [8] where rapid clearance is the key feature, firocoxib may be an extremely important component of multimodal analgesia with particular reference to visceral analgesia.

### Repeat dose administration

The aim of the repeat dose study was to determine whether firocoxib accumulates in koala plasma after repeat administration. A calculated accumulation factor greater than 1.3 indicates that the xenobiotic is likely to accumulate, and this was seen in 1/3 male and 3/3 female koalas. Haematological and biochemical analytes in all six animals were within reference ranges at the start of the study. Only two out of six animals had repeat profiles following the repeat dose study with no significant differences between the initial and final analytes see S8 Table. Despite the measured firocoxib accumulation in the females, there were no observed adverse effects in any of the koalas 72 h after twice daily firocoxib administration.

### Limitations

The major limitation of this pilot study was the low number of animals used and consequently the study was underpowered to find significant differences in some of the pharmacokinetic indices between the males and females. However, the data shows a trend of reduced oral absorption and slower elimination of firocoxib and its M1 metabolite in the females compared to the males. It has been reported that increased concentrations of oestrogen and progesterone can inhibit hepatic drug metabolism which may lead to drug accumulation in women [45]. Sex differences have been recognised in some NSAID PK profiles including differing activity in metabolism enzymes as well as in PD efficacy [46]. A recent meloxicam PK study in Romanov sheep reported higher plasma concentrations, longer half-life and lower clearance in the female sheep compared to the males [47]. In humans, higher plasma concentrations of the NSAID naproxen have been found in women than men, increasing proportionately with women’s ages. This is thought to occur due to faster activity of CYP2C in men than women [48]. Bioequivalence studies have shown some subtle differences in C_max_ and AUC values between men and women for the NSAIDs diclofenac and ketoprofen: men have a lower C_max_, but the AUC is greater for women [49]. Further studies are warranted to confirm that the difference in firocoxib PK profile is significantly different between mature male and female koalas.

## Conclusions

Firocoxib has some very useful anti-inflammatory properties demonstrated in *in vivo* and *in vitro* studies in rats, dogs, cats and horses [24,33,37,50–53]. It has antipyretic and analgesic properties for osteoarthritis in animals as well as some evidence of visceral analgesia. Firocoxib is very lipophilic and therefore is likely to persist in tissues and have a longer duration of action than reflected in the plasma half-life. The harmonic mean plasma half-life of firocoxib in male and female koalas is at least two and a half times that of meloxicam [5]. Based on this study, firocoxib is recommended as a NSAID that can be administered to koalas at 5 mg/kg orally twice daily for six administrations, however the dose for females may need to be increased but accordingly, the duration of dosing should be only once daily. Future studies are warranted to confirm that the difference in firocoxib PK profile is significantly different between mature male and female koalas; and to confirm the duration of firocoxib’s anti-inflammatory and analgesic effects by observing the clinical response and changes in inflammatory biomarkers, in sick and injured koalas [54–59].

## Supporting information

S1 Table Weights, ages and sexes of koalas. (DOCX)

S2 Table Individual plasma trough firocoxib concentrations (ng/mL) at four time points (blood collected just prior to the next dose) during repeat oral dosing of firocoxib (5 mg/kg) every 12 h for three days. (DOCX)

S3 Table Harmonic and arithmetic means ± SD, median, (range) and coefficient of variation of male versus female firocoxib concentrations trough plasma concentrations at 24, 48 and 72 h in the repeat dosing study. (DOCX)

S4 Table Metabolite 1 peak area in mV, at each blood collection time-point for each koala, when 5 mg/kg of firocoxib was administered as a single oral dose. ND = not detectable.
(DOCX)

S5 Table Metabolite 2 peak area in mV at each blood collection time-point for each koala, when 5 mg/kg of firocoxib was administered as a single oral dose. ND = not detectable; NS = no sample available.
(DOCX)

S6 Table Individual M1 peak area (mV) at 24, 48 and 72 h (blood collected just prior to the next dose) during repeat oral dosing of firocoxib (5 mg/kg) every 12 h for three days. (DOCX)

S7 Table Individual M2 peak area (mV) at 24, 48 and 72 h (blood collected just prior to the next dose) during repeat oral dosing of firocoxib (5 mg/kg) every 12 h for three days. (DOCX)

S8 Table Haematological and biochemical analyte values for koalas (K2 and K6) prior to treatment (T = 0 h) and 12 h at the conclusion of repeated dosing (at 72 h after the first drug administration). *ZIMS Expected Local Test Results for *Phascolarctos cinereus*. (2025, May). Species360 Zoological Information Management System. Retrieved from http://zims.Species360.org.
(DOCX)

S9 Table Species comparisons on firocoxib pharmacokinetic indices C_max_, T_max_ and half-life (t1/2) after oral and intravenous administration. * Median value.
(DOCX)
