# Supplementary material for: Pharmacokinetic profile of oral firocoxib in the koala (Phascolarctos cinereus)
Source: PLoS One. 2025 Sep 30;20(9):e0332448. doi: 10.1371/journal.pone.0332448 (PMC12483202; doi:10.1371/journal.pone.0332448)
Supplement: S1 Table — (DOCX) [file pone.0332448.s001.docx]

| Koala | **K1** | **K2** | **K3** | **K4** | **K5** | **K6** |
| --- | --- | --- | --- | --- | --- | --- |
| Weight (kg) | 9.7 | 9.4 | 9.6 | 7.5 | 7.8 | 8.9 |
| Age (years) | 9.1 | 4.7 | 4.3 | 4.8 | 4.8 | 8.9 |
| Sex | Male | Male | Male | Female | Female | Female |
