# Supplementary material for: Pharmacokinetic profile of oral firocoxib in the koala (Phascolarctos cinereus)
Source: PLoS One. 2025 Sep 30;20(9):e0332448. doi: 10.1371/journal.pone.0332448 (PMC12483202; doi:10.1371/journal.pone.0332448)
Supplement: S2 Table — (DOCX) [file pone.0332448.s002.docx]

|  | Plasma firocoxib concentration (ng/mL) | | | | | |
| --- | --- | --- | --- | --- | --- | --- |
|  | Male | | | Female | | |
| Time (h) | K1 | K2 | K3 | K4 | K5 | K6 |
| 0 | 0 | 0 | 0 | 0 | 0 | 0 |
| 24 | 25.3 | 26.1 | 47.5 | 69.1 | 89.0 | 71.3 |
| 48 | 11.9 | 57.6 | 48.2 | 36.8 | 60.2 | 69.8 |
| 72 | 61.8 | 35.9 | 56.2 | 136.7 | 102.5 | 120.0 |
