# Supplementary material for: Pharmacokinetic profile of oral firocoxib in the koala (Phascolarctos cinereus)
Source: PLoS One. 2025 Sep 30;20(9):e0332448. doi: 10.1371/journal.pone.0332448 (PMC12483202; doi:10.1371/journal.pone.0332448)
Supplement: S3 Table — (DOCX) [file pone.0332448.s003.docx]

|  | Male | | | | | Female | | | | |
| --- | --- | --- | --- | --- | --- | --- | --- | --- | --- | --- |
| Time (h) | Harmonic mean ng/mL | Arithmetic mean  ng/mL | SD  ng/mL | Median  (Range)  ng/mL | CV % | Harmonic mean  ng/mL | Mean  ng/mL | SD  ng/mL | Median  (Range) | CV%  ng/mL |
| 24 | 30.3 | 33.0 | 12.6 | 26.1  (25.3 - 47.5) | 38.2 | 75.5 | 76.5 | 10.9 | 71.3  (69.1-89.08) | 14.3 |
| 48 | 24.6 | 39.2 | 24.1 | 48.2  (11.9 - 57.6) | 61.5 | 51.6 | 55.5 | 16.9 | 60.2  (36.8-69.6) | 30.5 |
| 72 | 48.5 | 51.3 | 13.6 | 56.2  (35.9 - 61.8) | 26.6 | 118.1 | 119.73 | 17.09 | 119.9  (102.5-136.7) | 14.27 |
