# Supplementary material for: Pharmacokinetic profile of oral firocoxib in the koala (Phascolarctos cinereus)
Source: PLoS One. 2025 Sep 30;20(9):e0332448. doi: 10.1371/journal.pone.0332448 (PMC12483202; doi:10.1371/journal.pone.0332448)
Supplement: S4 Table — ND = not detectable. (DOCX) [file pone.0332448.s004.docx]

| **Time (h)** | **K1** | **K2** | **K3** | **K4** | **K5** | **K6** |
| --- | --- | --- | --- | --- | --- | --- |
| 0 | ND | ND | ND | ND | ND | ND |
| 0.25 | ND | 2037 | 5068 | ND | ND | ND |
| 0.5 | 5700 | 3173 | 11221 | 5087 | ND | 2708 |
| 1 | 14306 | 8053 | 17994 | 14645 | 34869 | 3750 |
| 2 | 73536 | 26745 | 92570 | 71859 | 145729 | 10048 |
| 4 | 262089 | 272235 | 368923 | 300651 | 230099 | 61637 |
| 8 | 307127 | 345896 | 343787 | 369357 | 282185 | 57743 |
| 12 | 313610 | 230425 | 189786 | 245133 | 196552 | 46726 |
| 24 | 69395 | 1218 | 48759 | 51183 | 72610 | 19253 |
| 36 | 19353 | 14671 | 13452 | 16803 | 84032 | 8882 |
| 48 | 6498 | 5009 | 3602 | 4802 | 22688 | 2183 |
