# Supplementary material for: Pharmacokinetic profile of oral firocoxib in the koala (Phascolarctos cinereus)
Source: PLoS One. 2025 Sep 30;20(9):e0332448. doi: 10.1371/journal.pone.0332448 (PMC12483202; doi:10.1371/journal.pone.0332448)
Supplement: S5 Table — ND = not detectable; NS = no sample available. (DOCX) [file pone.0332448.s005.docx]

| **Time (h)** | **K1** | **K2** | **K3** | **K4** | **K5** | **K6** |
| --- | --- | --- | --- | --- | --- | --- |
| 0 | ND | ND | ND | ND | ND | ND |
| 0.25 | ND | ND | ND | ND | ND | ND |
| 0.5 | ND | ND | ND | ND | ND | ND |
| 1 | ND | ND | 1584 | ND | 6174 | NS |
| 2 | ND | 2421 | 4484 | 2715 | NS | ND |
| 4 | ND | 7752 | 17040 | 10314 | 4838 | 2277 |
| 8 | 9375 | 12080 | 19718 | 23451 | 8479 | 3263 |
| 12 | 9385 | 12919 | 14108 | 23008 | 9043 | 2272 |
| 24 | 5912 | 5794 | 5742 | 7950 | 5635 | 1827 |
| 36 | ND | 2908 | 2598 | 4063 | 5506 | ND |
| 48 | ND | ND | ND | ND | 3450 | ND |
