# Supplementary material for: Pharmacokinetic profile of oral firocoxib in the koala (Phascolarctos cinereus)
Source: PLoS One. 2025 Sep 30;20(9):e0332448. doi: 10.1371/journal.pone.0332448 (PMC12483202; doi:10.1371/journal.pone.0332448)
Supplement: S6 Table — (DOCX) [file pone.0332448.s006.docx]

|  | Peak area (mV) | | | | | |
| --- | --- | --- | --- | --- | --- | --- |
|  | Male | | | Female | | |
| Time (h) | K1 | K2 | K3 | K4 | K5 | K6 |
| 24 | 205879 | 160084 | 392984 | 780073 | 723521 | 225325 |
| 48 | 189216 | 223867 | 355549 | 669550 | 633625 | 303645 |
| 72 | 257609 | 189542 | 404791 | 895148 | 1013744 | 442144 |
