# Supplementary material for: Pharmacokinetic profile of oral firocoxib in the koala (Phascolarctos cinereus)
Source: PLoS One. 2025 Sep 30;20(9):e0332448. doi: 10.1371/journal.pone.0332448 (PMC12483202; doi:10.1371/journal.pone.0332448)
Supplement: S7 Table — (DOCX) [file pone.0332448.s007.docx]

|  | Peak area (mV) | | | | | |
| --- | --- | --- | --- | --- | --- | --- |
|  | Male | | | Female | | |
| Time (h) | K1 | K2 | K3 | K4 | K5 | K6 |
| 0 | 0 | 0 | 0 | 0 | 0 | 0 |
| 24 | 17160 | 13963 | 20811 | 35614 | 29928 | 9810 |
| 48 | 16668 | 17458 | 21292 | 44782 | 33770 | 20347 |
| 72 | 17253 | 22132 | 25134 | 54644 | 39582 | 22381 |
