# Supplementary material for: Pharmacokinetic profile of oral firocoxib in the koala (Phascolarctos cinereus)
Source: PLoS One. 2025 Sep 30;20(9):e0332448. doi: 10.1371/journal.pone.0332448 (PMC12483202; doi:10.1371/journal.pone.0332448)
Supplement: S8 Table — *ZIMS Expected Local Test Results for Phascolarctos cinereus. (2025, May). Species360 Zoological Information Management System. Retrieved from http://zims.Species360.org. (DOCX) [file pone.0332448.s008.docx]

|  | **K2** | | **K6** | |  |
| --- | --- | --- | --- | --- | --- |
| **Haematological parameters** | **T = 0 h** | **72 h** | **T = 0 h** | **72 h** | **Reference intervals *** |
| WBC *10^9 cells/L | 7.92 | 8.25 | 2.97 | 3.19 | 2.63-14.19 |
| HGB g/L | 108 | 93 | 103 | 78 | 71-154 |
| HCT % | 34 | 36 | 45 | 22 | 30-49 |
| Neutrophils % | 35 | 48.7 | 48 | 65.1 | 15.4-76.2 |
| Neutrophil count *10^9 cells/L | 2.77 | 4.01 | 1.43 | 2.08 | 0.96-4.62 |
| Lymphocytes % | 64 | 49.7 | 49 | 31.8 | 14.6-83.9 |
| Lymphocyte count *10^9 cells/L | 5.07 | 4.1 | 1.46 | 1.01 | 0.81-6.66 |
| Monocytes % | 1 | 1.5 | 2 | 2.3 | 0.9-9.1 |
| Monocyte *10^9 cells/L | 0.08 | 0.12 | 0.06 | 0.07 | 0.037-0.780 |
| Eosinophils % |  | 0.1 | 1 | 0.6 | 0.9-8.0 |
| Eosinophil count *10^9 cells/L |  | 0.01 | 0.03 | 0.02 | 0.03-0.52 |
| **Biochemical analytes** |  | | | | |
| Glucose mmol/L | 4.2 | 3.3 | 5.8 | 4 | 2.96-8.37 |
| BUN mmol/L | 2.3 | 1.6 | 1.4 | <0.7 | 0.3-5.9 |
| Creatinine µmol/L | 80 | 102 | 86 | 150 | 46-154 |
| Ca mmol/L | 2.8 | 2.85 | 2.65 | 2.68 | 2.2-2.9 |
| Phos mmol/L | 1.43 | 1.48 | 1.43 | 1.37 | 0.87-2.47 |
| Na mmol/L | 141 | 142 | 135 | 139 | 132-143 |
| K mmol/L | 4.2 | 4.8 | 3.9 | 4.8 | 3.4-5.5 |
| Total protein (R) g/L | 62 | 62 | 64 | 65 | 52-78 |
| Total protein g/L | 61 | 62 | 61 | 61 | 56-76 |
| Albumin unspecified g/L | 38 | 39 | 41 | 43 |  |
| Globulin g/L | 23 | 23 | 20 | 18 | 18-34 |
| ALT U/L | 7 | 8 | 7 | 10 | 7.0-23 |
| Tot. Bili. µmol/L | 3 | 3 | 4 | 3 | 0.5-4.0 |
| Amylase U/L | 63 | 59 | 48 | 47 | 32-108 |
| Alkaline Phosphatase U/L | 51 | 63 | 56 | 49 | 54-941 |
