# Supplementary material for: Pharmacokinetic profile of oral firocoxib in the koala (Phascolarctos cinereus)
Source: PLoS One. 2025 Sep 30;20(9):e0332448. doi: 10.1371/journal.pone.0332448 (PMC12483202; doi:10.1371/journal.pone.0332448)
Supplement: S9 Table — * Median value. (DOCX) [file pone.0332448.s009.docx]

| Species (n) | **Route** | **Dose**  **(mg/kg)** | **C_max_**  **(ng/mL)** | **T_max_**  **(h)** | **t_1/2_**  **(h)** |
| --- | --- | --- | --- | --- | --- |
| Koala (6) | PO | 5 | 210.09 * (Male); 61.9* (Female) | 4.00* (both sexes) | 3.31* (Male); 7.24* (Female) |
| Dogs (40) [1] | PO | 5 | 520 | 1.25 | 5.9 |
| Dogs (6) [2] | PO | 5 |  |  | 6.5 |
| Horses (6) [1] | PO | 0.1 | 58.0 | 3.2 | 32.77 |
| Horses (12) [3] | PO | 0.1 | 75 | 3.9 | 30 |
| Horses (12) [3] | IV | 0.1 | 210 |  | 34 |
| Rabbits (6) [4] | PO | 3.74 -4.20 | 160 | 3.81 | 9.12 |
| Camels (5) [5] | IV | 0.1 |  |  | 5.75 |
| Pigs (7) [6] | PO | 4.0 | 60 | 7.41 | 24.075 |
| Calves (10) [7] | PO | 0.5 | 127.9 | 4.0 | 18.8 |
| Goats (10) [8] | IV | 0.5 |  |  | 26.41 |
| Goats (10) [8] | PO | 0.5 | 139 | 0.77 | 21.51 |
| Rhinoceros (5) [9] | PO | 0.1 | 15.7 | 4.0 | 4.96 |

**References**

1. Holland B, Fogle C, Blikslager A, Curling A, Barlow B, Schirmer J, et al. Pharmacokinetics and pharmacodynamics of three formulations of firocoxib in healthy horses. Journal of Veterinary Pharmacology and Therapeutics. 2015;38(3):249-56.

2. Morris T, Paine S, Zahra P, Li E, Colgan S, Karamatic S. Pharmacokinetics of carprofen and firocoxib for medication control in racing greyhounds. Australian veterinary journal. 2020;98(12):578-85.

3. Kvaternick V, Pollmeier M, Fischer J, Hanson P. Pharmacokinetics and metabolism of orally administered firocoxib, a novel second generation coxib, in horses. Journal of Veterinary Pharmacology and Therapeutics. 2007;30(3):208-17.

4. Gardhouse S, Kleinhenz M, Hocker SE, Weeder M, Montgomery SR, Zhang Y, et al. Pharmacokinetics and ex vivo pharmacodynamics of oral firocoxib administration in New Zealand White rabbits (Oryctolagus cuniculus). American Journal of Veterinary Research. 2022;83(7).

5. Wasfi IA, Saeed HM, Agha B, Kamel AM, Al Biriki NA, Al Neaimi KM, et al. Pharmacokinetics and metabolism study of firocoxib in camels after intravenous administration by using high-resolution bench-top orbitrap mass spectrometry. Journal of Chromatography B. 2015;974:17-23.

6. Kleinhenz MD, Odland C, Williams TE, Zhang Y, Fitzgerald AH, Sidhu PK, et al. Pharmacokinetics and tissue concentrations of firocoxib in sows following oral administration. Journal of Veterinary Pharmacology and Therapeutics. 2020;43(5):491-8.

7. Stock ML, Gehring R, Barth LA, Wulf LW, Coetzee JF. Pharmacokinetics of firocoxib in preweaned calves after oral and intravenous administration. Journal of Veterinary Pharmacology and Therapeutics. 2014;37(5):457-63.

8. Stuart AK, KuKanich B, Caixeta LS, Coetzee JF, Barrell EA. Pharmacokinetics and bioavailability of oral firocoxib in adult, mixed‐breed goats. Journal of Veterinary Pharmacology and Therapeutics. 2019;42(6):640-6.

9. Bryant B, Campbell-Ward M, Kimble B, Govendir M. Pharmacokinetic profiles of oral phenylbutazone, meloxicam, and firocoxib in Southern Black Rhinoceros (*Diceros bicornis minor*). Journal of Zoo and Wildlife Medicine. 2024;55(3):547-54.
